# Mechanism-based approach in designing patient-specific combination therapies for nonsense mutation diseases

**DOI:** 10.1093/nar/gkaf216

**Published:** 2025-03-29

**Authors:** Saleem Y Bhat, Arpan Bhattacharya, Hong Li, Xiaonan Cui, John D Lueck, Yale E Goldman, Barry S Cooperman

**Affiliations:** Department of Chemistry, University of Pennsylvania, Philadelphia, PA 19104, United States; Department of Chemistry, University of Pennsylvania, Philadelphia, PA 19104, United States; Department of Physiology, Perelman School of Medicine, University of Pennsylvania, Philadelphia, PA 19104, United States; Department of Chemistry, University of Pennsylvania, Philadelphia, PA 19104, United States; Department of Physiology, Perelman School of Medicine, University of Pennsylvania, Philadelphia, PA 19104, United States; Department of Pharmacology and Physiology, University of Rochester School of Medicine and Dentistry, Rochester, NY 14642, United States; Department of Physiology, Perelman School of Medicine, University of Pennsylvania, Philadelphia, PA 19104, United States; Department of Chemistry, University of Pennsylvania, Philadelphia, PA 19104, United States

## Abstract

Premature termination codon (PTC) diseases account for ∼12% of all human disease mutations. Although there are no FDA approved treatments for increasing PTC readthrough, one readthrough inducing drug, ataluren, has conditional approval for treatment of Duchenne muscular dystrophy elsewhere. Ataluren displays low toxicity in clinical trials for treatment of PTC diseases, but its therapeutic effects are inconsistent. The messenger RNA (mRNA) sequence context of a PTC is a major determinant of PTC readthrough efficiency. We have shown that ataluren stimulates readthrough exclusively by competitively inhibiting release factor complex (RFC) catalysis of translation termination. Here, using an *in vitro* reconstituted system, we demonstrate that PTC identity and the immediately adjacent mRNA sequence contexts modulate RFC activity in terminating peptide elongation. Such modulation largely determines the effectiveness of ataluren in stimulating readthrough, whether added alone or in combination with either the aminoglycoside G418 or an anticodon edited aa-tRNA, which stimulate readthrough by mechanisms orthogonal to that of ataluren. Our results suggest a potential rationale for the variability of ataluren effectiveness in stimulating readthrough. We hypothesize that patients harboring a PTC mutation within a sequence context promoting strong interaction with RFC will be resistant to ataluren, but that ataluren treatment will be more effective for patient sequences conferring weaker interaction with RFC.

## Graphical Abstract

**Graphical Abstract:**
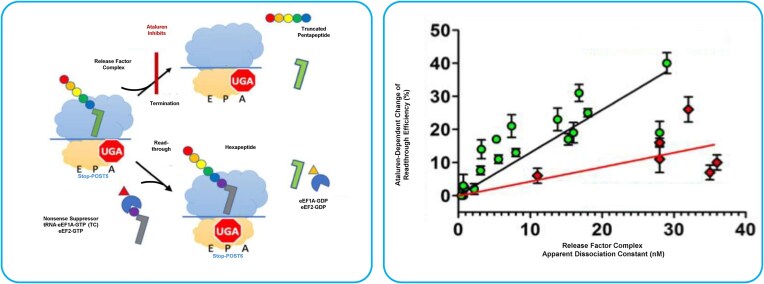

## Introduction

Premature termination codon (PTC) diseases, arising as a consequence of nonsense mutations in a patient’s DNA, account for ∼12% of all human disease mutations, including those giving rise to cystic fibrosis (CF), Duchenne muscular dystrophy (DMD), Marfan syndrome, and several cancers [1–4]. Despite their prevalence, there are currently no US Food and Drug Administration - approved treatments for increasing PTC readthrough in nonsense mutation diseases. The situation is similar worldwide, although since 2014, the European Medicines Agency and several other national regulatory agencies have given conditional approval of the translational readthrough inducing drug (TRID) ataluren (also known as PTC124 and marketed as Translarna) for treatment of DMD, the only such TRID approved for treatment of any PTC disease. Ataluren has been shown to stimulate readthrough of a PTC, resulting in the insertion of one of several different amino acids, depending on the identity of the pathological nonsense codon (UGA: W, R, or C; UAA or UAG: Q, K, or Y) [5–8]. Ataluren displays consistent low toxicity in clinical trials for treatment of several different PTC diseases, but its therapeutic effects on such diseases are inconsistent [9].

The lack of treatments for PTC diseases, which increase readthrough, has spurred research to develop clinically relevant nonsense suppressors [10–14]. These include small organic molecule TRIDs, anticodon-edited suppressor tRNAs (ACE-tRNAs), which have recently been shown to promote readthrough of disease-causing PTC mutations to an impressive extent in both cellular and animal studies [15–20], and messenger RNA (mRNA) and DNA editing [21, 22]. Another active field of research focuses on how the identity of the stop codon and its sequence context affect readthrough efficiency (RE), in both the absence and presence of nonsense suppressors [23–27]. It is clear from these studies, mostly in cells or cell extracts, that readthrough is most pronounced with the UGA PTC and that both downstream and upstream sequences significantly affect RE, although, for given sequence contexts, the magnitudes of RE enhancements differ considerably in the presence of different nonsense suppressors [23]. Recent results of Toledano *et al.* [23] and Mangkalaphiban *et al.* [24, 25] provide strong evidence that, in addition to the identity of the PTC itself (UGA, UAA, or UAG), by far the strongest effects of the nearby sequence context depend on one and possibly two codons immediately downstream (nt +4 to +9) and one codon upstream (nt −1 to −3) of the stop codon (nt +1 to +3). The downstream context is the more consequential [23–25, 28], likely reflecting in part variation in the direct interaction strength of the +4 nt, and possibly the +5 nt, with eukaryotic release factor 1 (eRF1) [29]. The upstream codon also plays a substantial role, with both Toledano *et al.* [23] and Mangkalaphiban *et al.* [24, 25] concluding that the identity of the P-site peptidyl-tRNA bound to this codon is primarily responsible for upstream effects on readthrough. Lesser effects result from variation in nt −4 to −6.

**Figure 1.**
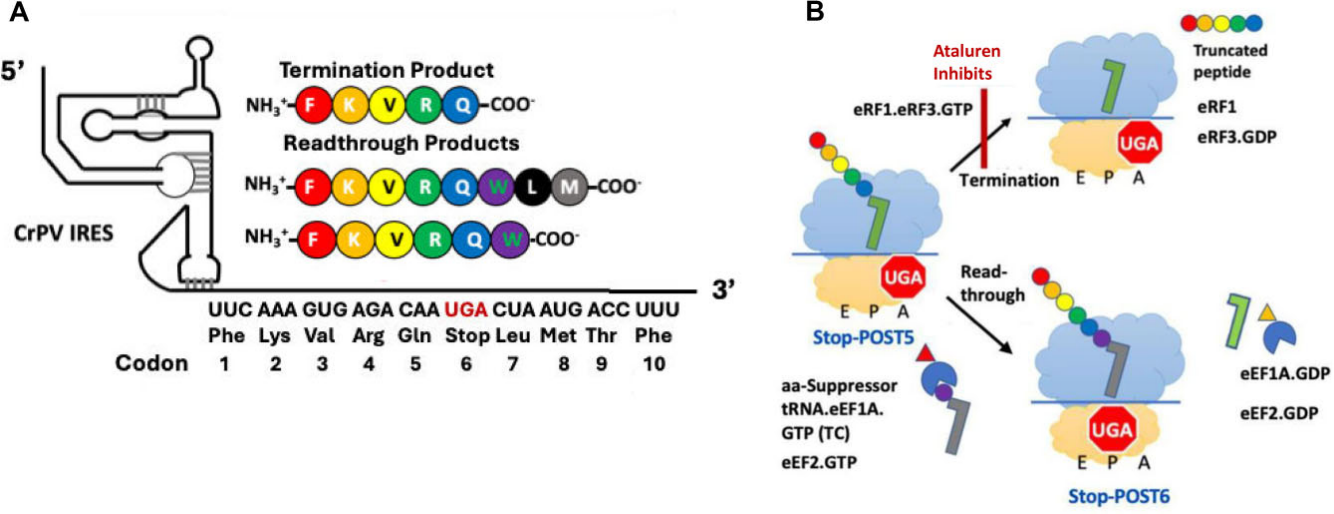
(**A**) The Pure-LITE system for measuring termination and readthrough. (**B**) RFC and suppressor TC compete for reaction with Stop-POST5 pretermination complex. For readthrough, Trp-tRNA^Trp^ is used as suppressor aminoacyl-tRNA.

Our interest in developing potent and clinically useful treatments of PTC diseases has led us to focus on ataluren, because of its low toxicity and its conditional approval for treatment of DMD. Recently, we developed a reconstituted *in vitro* eukaryotic system, denoted PURE-LITE, to conduct detailed mechanistic studies of eukaryotic protein synthesis [30–32]. PURE-LITE takes advantage of the ability of the intergenic internal ribosome entry site (IRES) of cricket paralysis virus (CrPV-IRES) to form a tight complex with 80S ribosomes, which is then capable of initiating cell-free synthesis of complete proteins in the absence of the complex set of natural initiation factors [33]. This allows for elongation and termination to be studied with the addition of just four factors, eEF1A and eEF2 for elongation and eRF1 and eRF3 for termination (Fig. 1A). Using the PURE-LITE system, we recently reported that ataluren stimulates readthrough exclusively by competitively inhibiting release factor complex (RFC, eRF1.eRF3.GTP)-dependent catalysis of translation termination, and does so via binding to at least two sites on the ribosome and possibly one on the RFC [32]. It thus has a mode of action orthogonal to that of the aminoglycoside G418, which, on binding to its high affinity site near the ribosome decoding site, stimulates readthrough by facilitating productive binding of near-cognate suppressor tRNAs without affecting termination activity [31] (Fig. 1B).

Here we present results demonstrating that (i) RFC termination activity has a strong dependence on the sequence context of PTCs found in patient mRNA sequences encoding CF transmembrane conductance regulator (CFTR) and fibrillin1 (FBN1), causing CF and Marfan syndrome, respectively; (ii) sequence dependent RFC activity correlates significantly with ataluren inhibition of termination and stimulation of readthrough; and (iii) combinations of ataluren and either of the nonsense suppressors ACE-tRNA^Arg^ and G418 have additive and occasionally synergistic effects on readthrough.

## Materials and methods

### Preparation of ribosomes, elongation factors, and aa-tRNAs

Stop-POST5 complexes listed in Tables 1 and 2 and used in termination and readthrough assays were prepared from shrimp (*Artemia salina*) 80S via a high KCl treatment of 80S ribosomes programmed with CrPV-IRES-mRNAs and the ternary complexes (TCs; aa-tRNA.eEF1A.GTP) of the appropriate aminoacyl-tRNAs, as described previously for preparing Reference Stop-POST5 [32]. *Saccharomyces cerevisiae* elongation factors eEF1A [34] and eEF2 [31, 35] and human eRF1 and eRF3 termination factors were prepared as reported previously [36, 37]. In the initial phases of this work, yeast tRNA^Phe^ was purchased from Sigma–Aldrich and other isoacceptor transfer RNAs (tRNAs) were prepared from bulk tRNA (Roche) from either *Escherichia coli* (tRNA^Val^, tRNA^Lys^, tRNA^Gln^, tRNA^Cys^, and tRNA^Met^) or yeast (tRNA^Arg^, tRNA^Trp^, tRNA^Glu^, tRNA^Lys^, tRNA^Thr^, and tRNA^Leu^) via hybridization to immobilized complementary oligoDNAs presented in Supplementary Table S1, as described previously [30, 38–40]. More recently we have prepared bulk yeast-tRNA from fresh Red Star yeast cakes as described [41]. ACE-tRNA^Arg^_UCA_ was prepared as described [15]. All tRNAs were charged with their cognate amino acids at 37°C using crude synthetase preparations from either *E. coli* or yeast which matched the source of the tRNA [38, 42, 43]. *S. cerevisiae* synthetase preparation was used to prepare Arg-ACE-tRNA^Arg^_UCA_. Atto 647-ϵ-lysine tRNA^Lys^, needed for the preparation of the Atto647-labeled Stop-POST5 complexes utilized in the fluorescence anisotropy termination assays, was prepared as described [32]. Cy5-heRF1 was prepared as described [44] by inserting a short peptide tag ybbR (DSLEFIASKLA) between the tobacco etch virus (TEV) protease cleavage site and the open reading frame using a QuickChange mutagenesis kit (Agilent) and then transformed into BL21(DE3) codon plus (Agilent) strain in the presence of ampicillin. Single colonies were grown overnight at 37°C in 100 ml of LB-amp media. Overnight cultures were diluted to 0.1 A_600nm_ and grown to 0.6 A_600nm_ in 2 l LB Amp media at 16°C. 0.5 mM IPTG was added, and the cell cultures were incubated at 16°C overnight. Cells were collected by centrifugation at 2700× *g* (4000 rpm in a GS3 rotor) for 20 min at 4°C. Cell pellet (∼5 g) was resuspended in eRF1/eRF3 equilibration buffer (100 mM HEPES-KOH, pH 7.4, 100 mM NaCl, 10% glycerol, 1 mM DTT, 50 ml) and lysed using a Qsonica sonicator at 30% for 10 15-s-pulse cycles with 30 s cooling on ice. Cell debris was spun down at 27 000× *g* (15 000 rpm in an SS34 rotor) for 15 min at 4°C. Cell lysate (∼50 ml) was loaded onto a 1.5 ml (3 ml slurry) TALON Superflow resin (Clontech) pre-equilibrated with equilibration buffer. The resin was washed three times with a 7.5 ml wash buffer (100 mM HEPES-KOH, pH 7.4, 10 mM imidazole, 100 mM NaCl, 10% glycerol, 1 mM DTT) and proteins were eluted in 0.5–1 ml fractions with elution buffer (100 mM HEPES-KOH, pH 7.4, 200 mM imidazole, 100 mM NaCl, 10% glycerol, 1 mM DTT). Fractions with ybbR tagged heRF1 were dialyzed against the storage buffer (100 mM HEPES-KOH, pH 7.6, 100 mM NaCl, 10% glycerol, 1 mM DTT) overnight and buffer exchanged into labeling buffer (50 mM HEPES, 10 mM MgCl_2_, and 1 mM DTT). For the labeling reaction, ybbR-heRF1 (10 μM, estimated using an e_280_ of 32 110 M^−1^ cm^−1^) was incubated with CoA-Cy5 (SiChem, Bremen) at a 4:1 dye-to-protein ratio in the presence of 4 μM Sfp enzyme (a kind gift from the Christian Kaiser lab) at room temperature for 45 min with continuous shaking. Excess dye was removed by passing the reaction mixture through a *P*-30 Bio Spin column (Bio-Rad). The labeled protein, having a labeling stoichiometry of ∼73%, was buffer exchanged into storage buffer (100 mM HEPES, 100 mM NaCl, 10% Glycerol, 1 mM DTT).

**Table 1.** Termination assay results

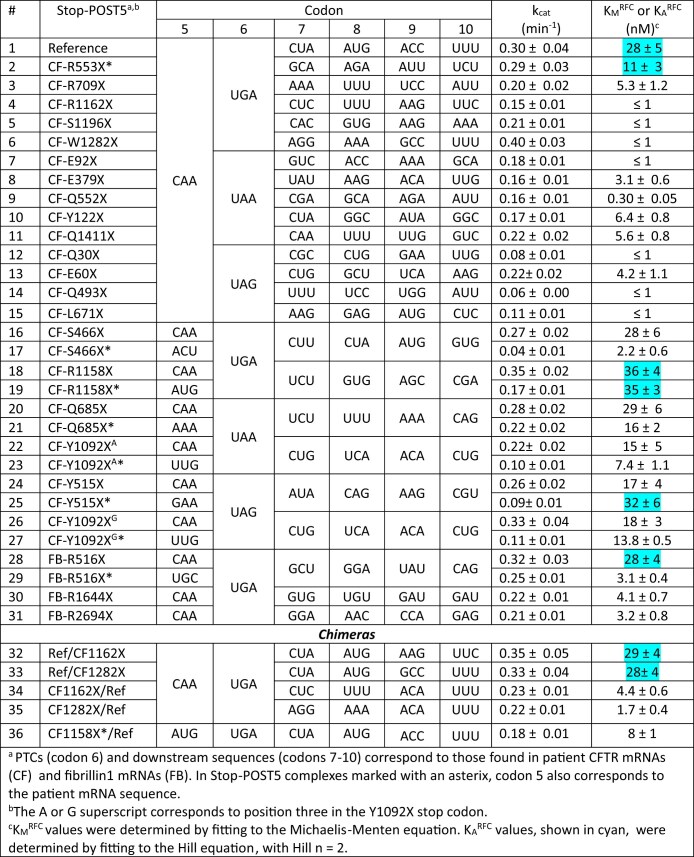

**Table 2.** Ataluren effects on termination^a^ and readthrough^b^

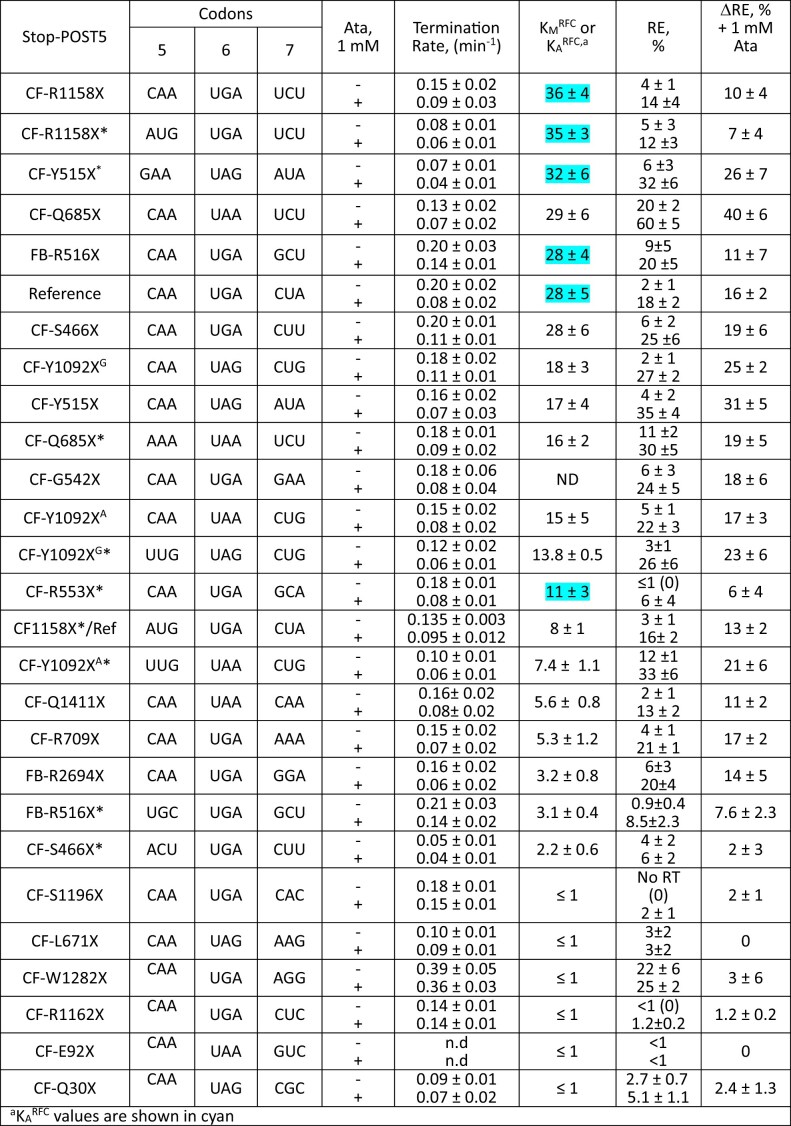

### Preparation of CrPV-IRES-variants

All CrPV-IRES-variant plasmid clones were obtained from Twist Biosciences and subsequently converted into the 309 residues long CrPV-IRES-mRNA by the HiScribe^®^ T7 high yield RNA synthesis kit (New England Biolabs, Catalog #E2040S) for use in the preparation of 80S. Stop-IRES and Stop-POST5 complexes. Shown below is the reference CrPV-IRES-mRNA sequence. The underlined residues mark the beginning of the attached mRNA moiety, with the bolded residues encoding the five amino acids upstream of the stop codon UGA (larger font), and the bolded, italicized residues encoding the four amino acids downstream from UGA.

GGAUCCUAAUACGACUCACUAUAGGGAGACCGGAAUUCAAAGCAAAAAUGUGATCUUGCUUGUAAAUACAAUUUUGAGAGGUUAAUAAAUUACAAGUAGUGCUAUUUUUGUAUUUAGGUUAGCUAUUUAGCUUUACGUUCCAGGAUGCCUAGUGGCAGCCCCACAAUAUCCAGGAAGCCCUCUCUGCGGUUUUUCAGAUUAGGUAGUCGAAAAACCUAAGAAAUUUACCU**UUCAAAGUGAGACAA**UGA*CUAAUG ACAUUU*CAAGAUACCAUGGAAGACGCCAAAAACAUAAAGAAAGGCCCGGAAGCUU.

Other CrPV-IRES-mRNA contexts, with sequences drawn mostly from CFTR (cystic fibrosis) and FBN1 (Marfan syndrome) patients harboring PTCs had the sequence design shown below.


\begin{eqnarray*}
- {\mathrm{ NN}}{{\mathrm{N}}_1}--{\mathrm{PTC}}--{\mathrm{NN}}{{\mathrm{N}}_2}--{\mathrm{NN}}{{\mathrm{N}}_3}--{\mathrm{NN}}{{\mathrm{N}}_4} \nonumber\\ --{\mathrm{NN}}{{\mathrm{N}}_5}
\end{eqnarray*}

The NNN_1_ upstream codon (codon 5 in Table 1) was either held constant as CAA, encoding tRNA^Gln^_UUG_, or variable, matching what is found in the patient sequence. PTC (codon 6 in Table 1) is the stop codon (UGA/UAG/UAA) and NNN_2_ to NNN_5_ are downstream codons (codons 7–10 in Table 1) mostly reproducing patient sequences. All IRES sequences were verified by sequencing performed in the University of Pennsylvania Genomic and Sequencing Core Facility.

### Preparation of stop-POST5 complexes

With one exception, all Stop-POST5 complexes were prepared by adding all five aminoacyl-tRNAs in a single step to CrPV-IRES-mRNA programmed 80S ribosomes in Buffer 4 (40 mM Tris–HCl, pH 7.5, 80 mM NH_4_Cl, 5 mM Mg(Ac)_2_, 100 mM KOAc, and 3 mM β-mercaptoethanol) in the presence of eEF1A, eEF2, and GTP as previously described [30, 32, 37]. The exception is the Atto647-labeled CF-Q685X*Stop-POST5 complex used in termination studies, which contains a Lys residue at both codons 2 and 5 (#21, Table 1) and was prepared in two steps. First, by addition of Phe-tRNA^Phe^ and Atto647-ϵ-lysine tRNA^Lys^ in the presence of eEF1A and eEF2 and GTP, and isolation of the ribosome bound Atto647-labeled Phe-Lys-tRNA^Lys^ by sedimentation through a 1.1 M sucrose cushion (120 min at 110 000 rpm using a S52-ST rotor on a Sorvall Discovery M120 SE ultracentrifuge). The resulting pellet was washed twice with Buffer 4 to remove any unbound Atto647-ϵ-lysine tRNA^Lys^, resuspended in Buffer 4, and centrifuged at 14 000 rpm for 30 min using a fixed angle F-45-30-11 rotor on an Eppendorf 5417C centrifuge. In the second step, Val-tRNA^Val^, Arg-tRNA^Arg^, and unlabeled Lys-tRNA^Lys^ were added in the presence of eEF1A, eEF2, and GTP to complete preparation of the Atto647-labeled CF-Q685X* Stop-POST5 complex.

### Termination assay

Termination assays were performed at 25°C in Buffer 4 with a TECAN SPARK multimode plate reader equipped with a monochromator, either in a 96-well (96 well Greiner^®^) or a 384-well (Corning) black flat bottom plate as described [32, 37]. Two solutions were prepared in Buffer 4, one containing 0.05 μM Atto(pep)-Stop-POST5 and 1 mM GTP and the other containing eRF1 (0–0.12 μM), eRF3 (0.8 μM), and 1 mM GTP in Buffer 4. Reaction was initiated by mixing the two solutions. All concentrations listed are final after mixing. The decrease in fluorescence anisotropy on release of the Atto647-labeled pentapeptide from the ribosome following eRF1-catalyzed hydrolysis of ribosome-bound pentapeptidyl-tRNA was monitored continuously. Excitation and emission wavelengths are preset in the plate reader for Atto-647. For measurements involving ataluren, both the RFC and Atto(pep)-Stop-POST5 solutions contained 1 mM ataluren. Time-dependent decreases in fluorescence anisotropy were analyzed with Graphpad Prism, using the single exponential with an asymptote model to obtain peptide dissociation rates for all Stop-POST5 complexes except for CF-W1282X, the results for which required a biphasic equation. The fast phase, corresponding to ∼50% of the release, was used to quantify the peptide release rate. All rate data were fit to the Michaelis–Menten or Hill equations to determine values of *k*_cat,_ and *K*_M_^RFC^ or *K*_A_^RFC^.

For most of the mRNA sequences, termination rate, *k*_obs_, plotted versus free RFC concentration (RFC_free_), was well fit by the Michaelis–Menten equation. Yielding maximum dissociation rate, *k*_cat_ and the half-saturation concentration of free RFC, *K*_M_^RFC^. For eight of the sequences, marked in cyan in Table 1, the plots of *k*_obs_ versus RFC_free_ were S-shaped and were fit by the Hill equation (equation 1), with *n* = 2, yielding *k*_cat_ and *K*_A_^RFC^.


(1) \begin{eqnarray*}
{k_{{\mathrm{obs}}}} = \;\frac{{{k_{{\mathrm{cat}}}}\; \cdot {\rm RFC}_{{\mathrm{free}}}^n}}{{{\rm RFC}_{{\mathrm{free}}}^n\; + \;{{\left( {K_{\mathrm{A}}^{{\mathrm{RFC}}}} \right)}^n}}}.
\end{eqnarray*}

The Hill equation reduces to the Michaelis–Menten equation when *n* = 1 (equation 2)


(2) \begin{eqnarray*}
{\left( {K_{\mathrm{A}}^{{\mathrm{RFC}}}} \right)^n} = \;\frac{{{\rm RFC}_{{\mathrm{free}}}^n \cdot {P_{{\mathrm{5\;free}}}}}}{{{\rm R}{{\rm P}_{\mathrm{5}}}}} = K_{\mathrm{M}}^{{\mathrm{RFC}}}{\mathrm{when\;}}n{\mathrm{\;}} = {\mathrm{\;}}1.
\end{eqnarray*}

RFC_free_ was calculated from the known total concentrations of RFC (i.e. RFC_total_) and of Stop-POST5 complex, shown as *P*_5 total_ in equation 3. RP_5_ is the concentration of the RFC·Stop-POST5 complex.


(3) \begin{eqnarray*}
{P_{{\mathrm{5\;free}}}} = \;{P_{{\mathrm{5\;total}}}} - \;{\rm R}{{\rm P}_5}{\mathrm{\;and}}\;{\rm RFC}_{{\mathrm{free}}}^n = \;{\rm RF}{{\rm C}_{{\mathrm{total}}}} - n \cdot {\rm R}{{\rm P}_{\mathrm{5}}}. \nonumber\\
\end{eqnarray*}

For *n* = 1, combining these equations into one relationship yields a quadratic equation that was solved analytically for RFC_free_. For *n* = 2, combining the equations yields a cubic equation that was solved numerically for RFC_free_ using the “solver” plug-in in Excel. *k*_cat_ and *K*_M_^RFC^ or *K*_A_^RFC^ were adjusted to fit equation 1 to the termination rate data *k*_obs_ versus RFC_free_ with *n* = 1 or 2. All measurements were repeated three times. The data shown are averages of three measurements ± SD.

### RE assays

**Figure 2.**
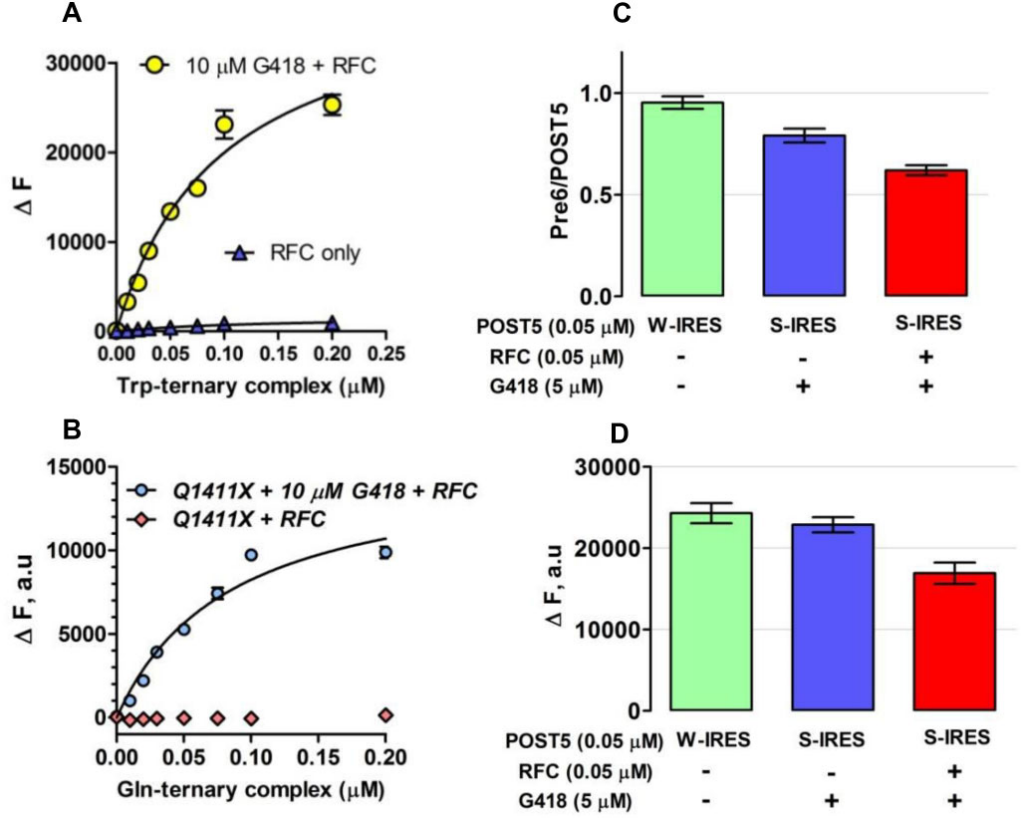
Readthrough assays. (**A**,**B**) Titration of proflavin-labeled suppressor aa-tRNA, with either tRNA^Trp(CCA)^ for UGA stop codon in Reference Stop-POST5 (Table 1, #1) and tRNA^Gln(UUG)^ for UAA stop codon in CF-Q1411X (Table 1, #11). (**C**,**D**) Comparing readthrough assay results. S-IRES refers to the Reference Stop-POST5 complex. W-IRES refers to a similar complex but with UGG, cognate to tRNA^Trp^ replacing UGA at codon 6. (C) Assay 1, measuring [^3^H] co-sedimenting with POST6 complexes. (D) Assay 2, measuring fluorescence increase, ΔF, accompanying formation of POST 6 complexes. Note that, in the absence of RFC, G418 induces almost complete readthrough of S-IRES, as previously demonstrated [31].

In RE assay 1, described earlier [31], we use co-sedimentation to determine the stoichiometry of [^3^H]-Stop-Pre6 complex formed when Stop-POST5 complex (0.05 μM) is incubated for 2 min @ 25°C, in Buffer 4, sufficient for essentially complete reaction, with [^3^H]-labeled suppressor aa-tRNA (0.2 μM), either near-cognate or cognate to the stop triplet in codon 6, in the presence of eEF1A (1 μM) and GTP (1 mM) and the absence or presence of eRF1 (0.05 μM) and eRF3 (0.2 μM). Near cognate suppressor tRNAs, Trp-tRNA^Trp^_CCA_, Tyr-tRNA^Tyr^_GUA_, and Gln-tRNA^Gln^_UUG_, added at 0.2 μM concentration, were used to measure readthrough at stop codons, UGA, UAG, and UAA, respectively. Reaction was initiated by mixing solution A containing Stop-POST5 complex with solution B containing TC ± RFC. All concentrations listed are final after mixing. When TRIDs were added, both solutions contained ataluren and G418, added either singly or in combination, at their final concentrations. Reactions were quenched with ice-cold 100 μl of 0.5 M MES buffer (pH 6.0) and placed on ice, 100 pmol of 70S carrier ribosomes were added, and ribosomes were collected by ultracentrifugation as previously described [31]. In RE assay 2, performed using a plate reader to measure fluorescence quantum yield increase during conversion of a proflavin-labeled Stop-POST5 complex to a proflavin-labeled Stop-POST6 complex. In this assay, the [^3^H]-labeled suppressor aa-tRNA is replaced by a proflavin-labeled suppressor aa-tRNA (0.1 μM), prepared as described [31], with either tRNA^Trp^_CCA_ or tRNA^Gln^_UUG_, having labeling stoichiometries of 2 and 1, respectively. All other conditions of reaction are as in RE assay 1. After mixing solution A with solution B, fluorescence was measured on the TECAN SPARK multimode reader for 10 min by exciting the fluorophore at 462 nm, and monitoring emission at 515 ± 15 nm. The value of Δ*F* was taken at 2 min, which corresponded to full reaction. The two assays gave similar results (Fig. 2C and D).

## Results

### Effects of sequence context on termination

#### Ensemble experiments

In our earlier use of PURE-LITE [31, 32, 37, 38], we prepared a pretermination complex, denoted here as Reference Stop-POST5, having a UGA stop codon at position 6, an upstream CAA codon at position 5, and the downstream sequence CUA AUG ACC UUU at codons 7–10 encoding L, M, T, F (Fig. 1A). The UGA stop codon, the upstream CAA codon, and the downstream CUA codon were chosen to favor readthrough [38]. The relatively high (20%) basal readthrough by near- cognate Trp-tRNA^Trp^, achieved by using an RFC concentration (typically ≤0.1 μM) much lower than that present in cells, permitted us to examine both termination and readthrough reactions. Readthrough was measured at codon 6 or 8 by incorporation into the nascent peptide of either [^3^H]-Trp or [^35^S]-Met, respectively. Here we extend these studies to first examine the effects on termination of varying (i)stop codon identity, (ii) four codons downstream of the stop codon, and (iii)one codon upstream of the stop codon, which, based on studies referred to above [23–25], include the mRNA contexts most consequential in determining REs. These variations necessitated the construction of a significant number of Stop-POST5 complexes, which, compared to Reference-Stop-POST5, can have changes in codons 5–10, as indicated in Table 1. The large majority of the Stop-POST complexes examined, 29/36 in Table 1, maintained CAA as the immediate codon upstream of the stop codon, to highlight differences due to downstream sequences, reflecting their known greater influence on RE [23–25, 28].

We used a plate-reader assay [32, 37] to determine the rates of termination for each of the Stop-POST5 complexes as a function of RFC concentration. This assay measures fluorescence anisotropy decrease as a fluorescent-labeled nascent peptide is released from the Stop-POST5 complex by the catalytic activity of RFC. The termination results for all Stop-POST5 complexes are presented in Table 1. The results for 28 of these Stop-POST5 complexes show a rectangular hyperbolic dependence on RFC concentration, corresponding to an apparent single effective site of RFC binding to the ribosome, fitting well to the Michaelis–Menten equation, and allowing quantification of *K*_M_^RFC^. In contrast, 8 Stop-POST5 complexes display a clear S-shaped dependence on RFC concentration, providing evidence for positive homotropic cooperativity in binding of RFC to the ribosome. *K*_A_^RFC^ values were determined for these sequences from the Hill equation (equation 1, see “Materials and Methods” section), after calculating free [RFC], taking into account RFC bound, and setting the Hill *n* = 2.

**Figure 3.**
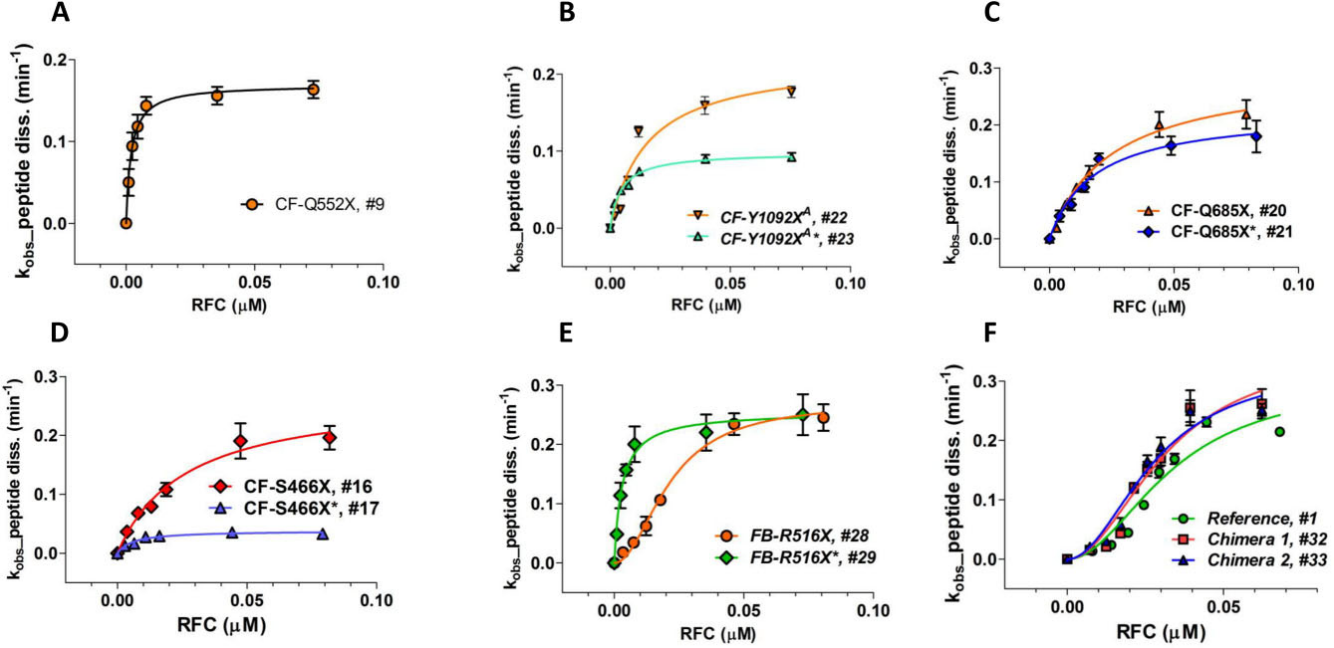
Representative results showing the dependence of the rate of peptide dissociation from Stop-POST5 complexes, numbered as in Table 1, on the free concentration of RFC. (**A**–**D**) Results fit to the Michaelis–Menten equation. (**B**–**D**) Variable effects due to changing the upstream codon 5 from CAA to the codon present in a patient mutation are shown. (**E**) Results fit to the Hill equation (#28) or the Michaelis–Menten equation (#29). (**F**) Results fit to the Hill equation, *n* = 2, for Reference (#1, circles), Chimera 1 (#32, squares), and Chimera 2 (#33, triangles).

Our lower limit for estimation of either *K*_M_^RFC^ or *K*_A_^RFC^, ≤1.0 nM, is determined by the lowest concentration of Stop-POST5 complex we employed, which gave a reliable fluorescence anisotropy decrease. Within this limitation, *K*_M_^RFC^ and *K*_A_^RFC^ values were broadly distributed over a ≥35-fold range, with all three of the stop codons. In contrast, *k*_cat_ values vary over a narrower range, ∼10-fold, and are not broadly distributed, with 90% falling between 0.1 and 0.3 min^−1^. Termination rates versus [RFC]_free_ for representative sequences are presented in Fig. 3. Four sequences for which the upstream codon (position 5, nts −1 to −3 relative to the stop codon) CAA encoding Gln is held constant (#s 9, 16, 20, and 22 in Table 1), show the clear dependence of *K*_M_^RFC^ on the downstream codons 7–10: ≤1 nM for # 9 (Fig. 3A) and higher for #s 22 (15 ± 5 nM), 20 (24 ± 3 nM), and 16 (26 ± 5 nM) (Fig. 3B–D, respectively). Changing the upstream codon from CAA, which generally favors readthrough [23], to the codon present in each patient sequence, has variable effects (Table 1). For the seven pairs of Stop-POST complexes examined, *k*_cat_ either shows little change (sequence #s 20, 21; 28, 29) or substantial decrease for the disease sequences (2- to 6-fold, #s 16, 17; 18, 19; 22, 23; 24, 25; 26, 27). Greater variability is seen in the interaction of RFC with Stop-POST5 as measured by either *K*_M_^RFC^ or *K*_A_^RFC^, which can show little change (#s 18,19; 20, 21; 26, 27), significant decrease (3- to 10-fold: #s 16, 17; 22, 23; 28, 29), or significant increase (2-fold, #s 24, 25). Interestingly, replacing the upstream CAA at codon 5 with the patient codon can result in a change in RFC concentration dependence from S-shaped to a rectangular hyperbola (Fig. 3E, #29 versus #28) or vice-versa (#25 versus #24). In order to determine the relative effects on termination activity of downstream codons 7 and 8 versus 9 and 10, we examined Stop-POST5 complexes derived from four chimeric mRNAs. In Ref/CF-1162X (#32) and Ref/CF1282 (#33), codons 7 and 8 are derived from Reference Stop-POST5 (#1), which has an S-shaped dependence on RFC concentration and a *K*_A_^RFC^ of 28 ± 5 nM. In contrast, codons 9 and 10, derived from CF-1162X (#4) and CF-1282X (#6), respectively, catalyze peptide release with a rectangular hyperbolic RFC concentration dependence, with *K*_M_^RFC^ values of ≤1 nM. In CF1162/Ref (#34) and CF1282/Ref (#35), the identities of the downstream codons are reversed. The termination activity results demonstrate clearly that the identities of the proximal downstream codons 7 and 8 largely determine the response of Stop-POST5 complexes to RFC, whereas the more distal downstream codons 9 and 10 matter less. Thus, as shown in Fig. 3F and Table 1, the dependence of termination on RFC concentration of the Reference (#1), Ref/CF-1162X (#32) and Ref/CF1282 (#33) Stop-POST5 complexes are almost identical with respect to both *K*_A_^RFC^ and *k*_cat_ whereas *K*_M_^RFC^ values for CF1162/Ref (#34) and CF1282/Ref (#35) are much closer to those found for CF-1162X (#4) and CF-1282X (#6), respectively, than to that found for Reference *K*_A_^RFC^.

#### smFRET experiments

**Figure 4.**
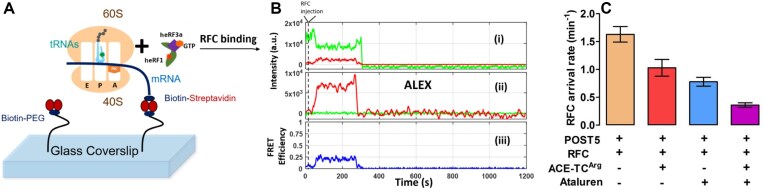
Sm FRET measurement of RFC interaction with Stop-POST 5 complexes. (**A**) Experimental design. (**B**) Sample traces measuring the time dependence for interaction of an RFC containing Cy5-heRF1 with Reference-Stop-POST5 complex containing FKVRQ-tRNA^Gln^(Cy3) bound in the P-site. These traces permit measurement of the rates of RFC arrival (*k*_arrival,app_), dissociation of tRNA^Gln^(Cy3), and dissociation of Cy5-heRF1 (*k*_dis,tRNA,app_) from, respectively, the times of ALEX (ii) or FRET (iii) appearance, Cy3 disappearance (i) and ALEX disappearance (ii). These values for 13 Stop-POST5 complexes are presented in Supplementary Table S2. (**C**) Effects of ataluren and ACE-tRNA, added separately or in combination, on *k*_arrival,app_.

RFC binding to a pretermination complex such as Stop-POST5 is followed by rapid release of eRF3 and by a much slower release of eRF1 [45]. We employed an single molecule fluorescence resonance energy transfer (smFRET) approach to determine whether the large range of *K*_m_^RFC^ and *K*_A_^RFC^ values as a function of sequence context shown in Table 1 is due either to differences in the rate of RFC binding to Stop-POST5 complexes (*k*_arrival,app_) or, following peptidyl-tRNA hydrolysis, in the rate of release from the ribosome of tRNA (*k*_dis,tRNA,app_) or eRF1 (*k*_dis,heRF1,app_). For this purpose we prepared a Cy5-labeled derivative of human eRF1 (Cy5-heRF1) and used it to form an active RFC complex with heRF3.GTP (Cy5-heRFC). Binding of Cy5-heRF1 to Stop-POST5 complexes, each of which contained tRNA-labeled FKVRQ-tRNA^Gln^(Cy3) bound in the P-site, adjacent to the stop codon, generated a transient FRET signal that disappears following peptidyl-tRNA hydrolysis and release of Cy5-heRF1 and tRNA^Gln^(Cy3). In these experiments, the Cy3-labeled Stop-POST5 complex was attached to a microscope cover slip chamber via the mRNA and a 3′ a biotin-streptavidin linkage (Fig. 4A). To initiate the binding reaction, Cy5-heRFC (35 nM) was injected into the flow cell 10 s after the video recording began (Fig. 4B). The arrival of Cy5-heRFC, detected directly using alternating laser excitation (ALEX) at wavelengths of 532 and 640 nm (panel ii, red trace), was closely followed by partial quenching of the Cy3-tRNA fluorescence (panel i, green trace) and an increase in the sensitized emission of Cy5 (panel i, red trace) due to FRET. The transient FRET efficiency of *E* = ∼0.25 between Cy5-heRF1 and Cy3-tRNA (panel iii, blue trace) is consistent with heRF1 being successfully accommodated within the ribosomal A site, allowing peptidyl-tRNA hydrolysis to proceed. Cy5-heRF1 and tRNA^Gln^(Cy3) were released with some delay after hydrolysis.

We performed the smFRET experiment on 13 different Stop-POST5 complexes. Apparent rate constants for RFC binding (*k*_arrival,app_) and both tRNA^Gln^ (*k*_,tRNA_^Gln^_,app_) and heRF1 (*k*_dis,heRF1,app_) release for each of the Stop-POST5 complexes, measured at a single RFC concentration (35 nM) are shown in Table S2. All *k*_arrival_,_app_ values fall within a two-fold range, as do 12 of the 13 values of *k*_,tRNA,app_ and *k*_dis,heRF1,app_, contrasting sharply with the ≥30–40 fold difference in measured *K*_M_^RFC^ and *K*_A_^RFC^ values for the same Stop-POST5 complexes. One exception to the limited range of these data is CF-R553X*, for which *k*_dis,tRNA,app_ and *k*_dis,heRF1,app_ are four- to five-fold slower than the fastest ones. These results provide a clear indication that differences in *K*_M_^RFC^ and *K*_A_^RFC^ are not due to differences in the rates of RFC binding or of tRNA or eRF1 release. Possible later reaction steps that control termination rates and RFC activity are reversible RFC dissociation, eRF3 GTPase, P_i_ release, or eEF3.GDP dissociation. Further studies will be needed to determine which of these steps show a clear correlation with *K*_M_^RFC^/*K*_A_^RFC^ values.

Our results measuring the effects of PTC sequence context on termination activity show that the identities of the immediate upstream codon (codon 5) and immediate downstream codons (codons 7 and 8) can have strong effects on both the strength of RFC binding, as measured by *K*_M_^RFC^ or *K*_A_^RFC^, and the rate constant for peptide release at saturating RFC (*k*_cat_). We have not yet determined the underlying cause for the large spread of *K*_M_^RFC^ /*K*_A_^RFC^ values, but can rule out effects on the rates of RFC binding, and of tRNA or eRF1 release following peptidyl-tRNA hydrolysis as being responsible.

### Effects of sequence context on RE, as modified by addition of ataluren

**Figure 5.**
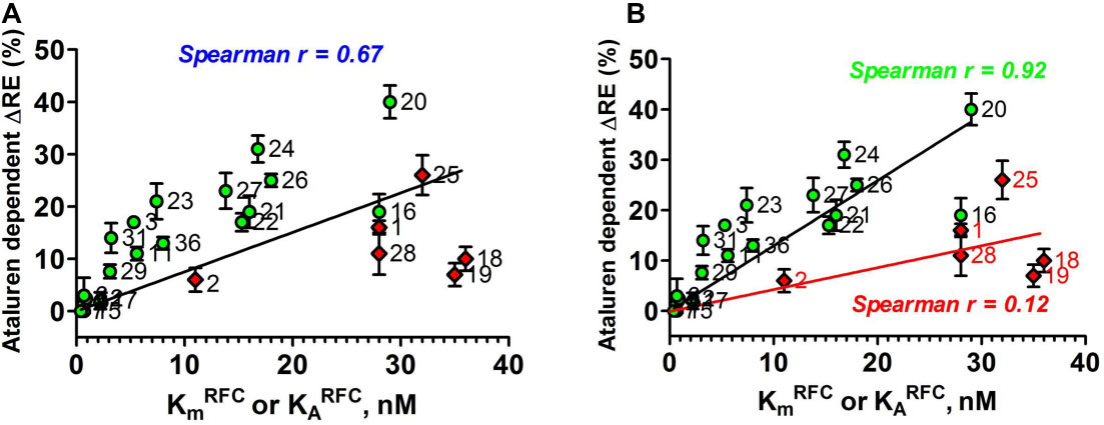
Correlation of ataluren stimulation of RE and *K*_M_^RFC^ (circles) and *K*_A_^RFC^ (diamonds) values. Stop-POST5 complexes are numbered as in Table 1. (**A**) All values are fit to a linear regression model. (**B**) *K*_M_^RFC^ and *K*_A_^RFC^ values are fit separately to linear regression models.

RE measured in the absence of nonsense suppressors reflects a competition between RFC and near-cognate TC for covalent reaction with Stop-POST5, i.e. hydrolysis of pentapeptidyl-tRNA versus elongation to hexapeptidyl-tRNA (Fig. 1B). We use two different assays to determine RE, which give equivalent results (Fig. 2C and D). Ataluren addition, by competitively inhibiting RFC binding [32] to Stop-POST5 without affecting near-cognate TC binding [31], alters the RFC versus TC competition in favor of the near-cognate TC, thereby stimulating readthrough. Ataluren effects, added at 1 mM, on termination and readthrough for 27 Stop-POST5 complexes are presented in Table 2. ΔRE values (± ataluren) generally increase as *K*_M_^RFC^/*K*_A_^RFC^ values increase, indicating weaker interaction of RFC with a Stop-POST5 complex, consistent with our expectations based on ataluren competitive inhibition of RFC. The Spearman correlation coefficient between these parameters is *r* = 0.67 (Fig. 5A). Because ΔRE values are consistently lower for *K*_A_^RFC^ values than for comparable *K*_M_^RFC^ values, we observe much stronger correlation (Spearman *r* = 0.92) between readthrough and termination effects when the 21 *K*_M_^RFC^ values are considered separately (Fig. 5B).

The ΔRE results in Table 2 were obtained at single concentrations of both ataluren (1 mM) and Suppressor-TC (Sup-TC, 0.2 μM), raising the possibility that outliers might arise from Stop-POST5 complexes differing in their dependencies on ataluren and Sup-TC concentrations. This does not appear to be the case, based on results presented in Supplementary Fig. S1A and B, showing that Stop-POST5 complexes having similar *K*_M_^RFC^/*K*_A_^RFC^ values but differing in ΔRE values have similar dependencies on ataluren and Sup-TC concentrations.

### Effects on RE of adding ataluren in combination with G418

**Figure 6.**
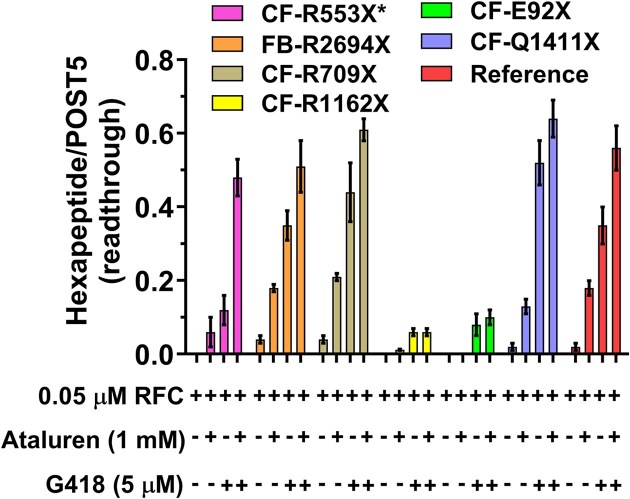
Ataluren and G418 promote readthrough of CFTR and FBN1 mutants either synergistically (CF-R553X*) or additively (four others). Ataluren has little or no effect in promoting readthrough of CF-R1162X or CF-E92X.

Added separately, both ataluren and G418 stimulate readthrough of Reference Stop-POST5 complex [31], leading to formation of FKVRQW-tRNA^Trp^. Combining ataluren and G418 leads to increased readthrough, as expected based on their orthogonal mechanisms for stimulating RE: i.e. ataluren inhibits termination without affecting binding of near-cognate aa-tRNA leading to peptide elongation, whereas G418 stimulates binding of near-cognate aa-tRNA leading to peptide elongation without affecting termination (Fig. 1B) [31]. Sample results presented in Fig. 6 show that effects of these combinations of nonsense suppressors can be additive (Stop-POST5 complexes Reference, CF-G542X, CF-R709X, CF-S446X) or synergistic (Stop-POST5 CF-R553X*). Neither CF-E92X nor CF-R1162X show ataluren stimulation of G418-induced readthrough, consistent with their tight RFC binding (Table 1, #s 4 and 7) and resistance of these Stop-POST5 complexes to readthrough stimulation by ataluren alone (Fig. 6).

### Effects on termination and RE of adding ataluren in combination with ACE-tRNA^Arg^_UCA_

**Figure 7.**
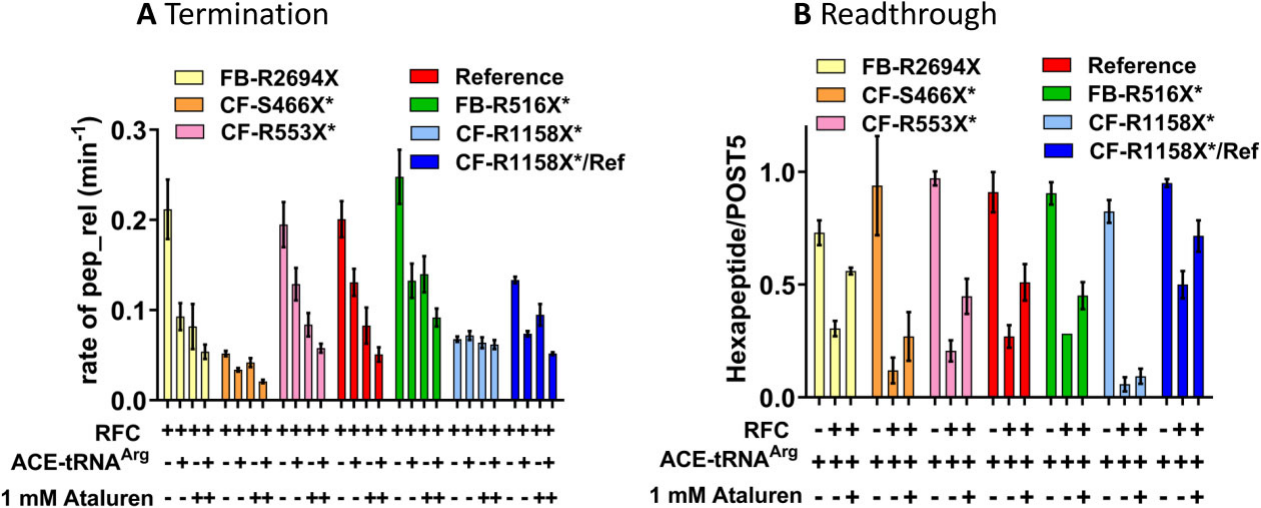
Ataluren acts consistently in its effect on ACE-tRNA^Arg^_UCA_ competition with RFC, as measured by termination (**A**) or readthrough (**B**). The six Stop-POST5 complexes showing additive effects of ataluren and ACE-tRNA^Arg^_UCA_ in inhibiting termination (A) also show partial reversal by ataluren of RFC inhibition of readthrough by ACE-tRNA^Arg^_UCA_ (B). Similarly, neither ACE-tRNA^Arg^_UCA_ nor ataluren show significant effects in either inhibiting termination or stimulating readthrough for the CF-R1158X* Stop-POST5 complex. Concentrations, ACE-tRNA^Arg^_UCA_ (0.2 μM), RFC (0.0625 μM).

Since TC is a molecular mimic of RFC [29], competing directly with it for binding to the A-site of the pretermination Stop-POST5 complexes used in this work (Fig. 1B), we expect that ACE-tRNA^Arg^_UCA_ will compete with RFC for binding to Stop-POST5 complexes with a UGA stop codon, leading to both inhibition of termination and directly forming the readthrough product. It has been proposed [46] that the identity of the peptidyl-tRNA bound in the P-site of a pretermination complex could affect this competition. To test this point, we determined ensemble termination rates and REs for seven different Stop-POST5 complexes in the presence of ACE-tRNA^Arg^_UCA_ and ataluren, added separately or in combination, giving the results displayed in Fig. 7A and B. In these experiments the ACE-tRNA^Arg^_UCA_ TC and RFC concentrations were chosen to make clear the effects of ataluren on the competition between these two complexes. All seven Stop-POST5 complexes, containing P-site bound FKVRZ-tRNA^Z^ (Z = Q, T, or M) gave consistent results between the termination and readthrough assays. Six sequences showed additive effects between ataluren and ACE-tRNA^Arg^_UCA_ both in inhibiting RFC activity and stimulating readthrough in the presence of RFC, leading to the formation of ribosome-bound FKVRZR-ACE-tRNA^Arg^_UCA_ (Z = Q or T). One sequence, CF-R1158X* (Z = M, Table 1, #19), resisted both ataluren (Table 2) and ACE-tRNA^Arg^_UCA_ inhibition of RFC activity (Fig. 7A), and ACE-tRNA^Arg^_UCA_ readthrough of the UGA stop codon (Fig. 7B). CF-R1158X* Stop-POST5 differs from Reference Stop-POST5 (Table 1, #1) in both its upstream codon 5 (AUG versus CUA) and its downstream codons, 7–10 (UCU GUG AGC CGA versus CUA AUG ACC UUU). The lack of responsiveness to ACE-tRNA^Arg^_UCA_ appears to be due mainly to the difference in the downstream sequence, since the chimeric CF-R1158X*/Ref Stop-POST5 (Table 1, #36), having the variable sequence AUG UGA CUA AUG ACC UUU, displays considerable ACE-tRNA^Arg^_UCA_ and ataluren inhibition of RFC activity (Fig. 7A) as well as ACE-tRNA^Arg^_UCA_ readthrough of the UGA codon and ataluren stimulation of such readthrough (Fig. 7B). These results indicate that ACE-tRNA^Arg^_UCA_ will generally support readthrough at a UGA stop codon and that such readthrough is enhanced by ataluren, but that some UGA stop codon sequence contexts, here exemplified by CF-R1158X*, can resist readthrough by ACE-tRNA^Arg^_UCA_.

We applied the smFRET approach described earlier (Fig. 4A and B) to determine the effects of adding ataluren and ACE-tRNA^Arg^_UCA_ on the interaction of Reference-Stop-POST5 with RFC. As expected, *k*_arrival,app_ is substantially decreased by each of these agents added alone, and is decreased significantly further when the agents are combined (Fig. 4C), consistent with the ensemble results presented in Fig. 7A. Neither agent has significant effects on *k*_heRF1 dis_, but each decrease *k*_tRNA_^Gln^_UUG_ by ∼40%. However, in this case the effects are not additive.

## Discussion

### Mechanism studies using the reconstituted *in vitro* PURE-LITE system

Here we demonstrate that PTC identity and mRNA sequence context modulate the catalytic activity of RFC in terminating peptide elongation, and, in so doing, determine the effectiveness of ataluren, a TRID that acts exclusively as a competitive inhibitor of RFC binding [31, 32], in stimulating RE. We see such stimulation by ataluren acting alone, or cooperatively in combination with either the aminoglycoside G418 or an ACE-tRNA. RE reflects the competition between productive binding of a suppressor TC (Sup-TC) and RFC to an A-site stop codon. This competition strongly favors RFC in general, since natural Sup-TCs, which are only near-cognate to the stop codons, bind more weakly than RFCs and, once bound, are subject to rejection by proofreading, which reduces the frequency of productive binding. Ataluren shifts the Sup-TC versus RFC competition in favor of the Sup-TC (Table 2), resulting in a significant correlation between sequence context effects on *K*_M_^RFC^/*K*_A_^RFC^ and ataluren stimulation of RE (Fig. 5A), which is considerably more marked when *K*_M_^RFC^ results are considered separately (Fig. 5B).

In contrast to these very clear relationships, our results raise three interesting questions, which will require further experiments to resolve. First, why do most of the Stop-POST5 complexes we studied (Table 1) show a hyperbolic dependence of termination rate on RFC concentration, consistent with RFC binding to the single, structurally well characterized site on the ribosome leading to termination [29, 47], while a minority show a sigmoidal dependence, indicating that RFC binding to an additional site (or sites) is required for their termination activity? The *K*_A_^RFC^ (sigmoidal curves) and *K*_M_^RFC^ (hyperbolic curves) have mean values of 28 and 9 nM, respectively (Table 2), suggesting that weaker interaction of RFC with the Stop-POST5 complex could be a necessary, although not sufficient condition for sigmoidal dependence. An hypothesis which would account for our results is that some RFC complexes which interact weakly with the well characterized RFC site on the ribosome have suboptimal orientations for termination activity which RFC binding to an additional ribosomal site (or sites) can correct, and binding to such sites is not influenced by ataluren. The P1 and P2 stalk of the ribosome could provide a possible location for such a site (or sites), to which one or more RFCs could bind via the eRF3 GTPase component of RFC [48].

Second, what accounts for the clear deviations from strict linearity in the plot of ΔRE versus *K*_M_^RFC^ (Fig. 5B), most evident in the two-fold ranges in ΔRE values for the Stop-POST5 complexes clustered at *K*_M_^RFC^ values between 5–7 and 15–19 nM? We think it likely that these deviations arise from steps in the overall process of termination following Sup-TC binding to the ribosomal A-site, which may not be sensitive toward ataluren. For example, it is clear from results presented in Fig. 3 that the identity of the P-site bound peptidyl-tRNA can strongly influence ΔRE, consistent with results of others [23–25, 49]. Also of possible relevance are results of Kolakada *et al.* [50] showing that the identity of P-site bound peptidyl-tRNA affects nonsense-mediated decay, which also competes with readthrough in cells. In addition, using an smFRET approach, we have recently shown that addition of the near-cognate Trp-TC to either the Reference Stop-POST5 complex (Table 1) or to a POST5 complex in which the UGA codon of Reference Stop-POST5 complex is replaced by the Cys codon UGU, requires several brief near cognate Trp-TC binding (sampling) events prior to stable binding leading to FKVRQW-tRNA^Trp^ formation [51]. Further, during the sampling period, a metastable ribosome conformation is formed, in which the accommodation step resulting in peptide elongation is retarded. Accumulation of this conformation may lead to ribosome stalling and the limited stoichiometry of peptide elongation observed when ribosomes interact with near-cognate TCs or inhibitory codon pairs [31, 52], and could certainly impact ΔRE values.

Third, how general a phenomenon is the resistance demonstrated by CF-R1158X*-Stop-POST5 to readthrough by ACE-tRNA^Arg^_UCA_ (Fig. 7B)? As discussed above, the stop codon and the immediate upstream codon and 1–2 downstream codons are considered to be the most consequential for nonsense suppressor stimulated readthrough [23–25]. In CF-R1158X*-Stop-POST5, this quartet of codons 5–8 has the sequence AUG UGA UCU GUG (Table 1, #19). The readthrough results obtained with CF-R1158X*/Ref-Stop-POST5 (Fig. 7B), which has the corresponding sequence AUG UGA CUA AUG (Table 1, #36), suggest that it might be the downstream UCU GUG codons that somehow reduce ACE-tRNA^Arg^_UCA_ RE, at least in this sequence, but perhaps more generally.

### Potential relevance for clinical studies

Ataluren has been investigated for its potential to readthrough disease causing PTCs in clinical trials, animals, and cell-based assays, with variable outcomes [9]. The results presented in this paper (Tables 1 and 2 and Figs 3 and 5–7), demonstrate the strong dependence on mRNA sequence context of ataluren-induced readthrough of PTCs, which is in turn strongly correlated with ataluren inhibition of RFC activity. As such, they provide an attractive hypothesis for the variability of ataluren effectiveness, suggesting that patients harboring a PTC mutation with a sequence context leading to very strong interaction with RFC (low *K*_M_^RFC^ or *K*_A_^RFC^ values) will likely be resistant to ataluren, whereas sequence contexts conferring weaker interaction with RFC (higher values of *K*_M_^RFC^ or *K*_A_^RFC^) would likely be more amenable to ataluren treatment, either added alone, or combined with other nonsense suppressors such as ACE-tRNAs or small molecule organic drugs, such as the aminoglycoside ELX-02 [53]. This hypothesis would be strengthened if the *in vitro* results reported here were found to be consistent with similarly targeted cellular studies examining ataluren stimulation of readthrough.

Another and highly salient test of the hypothesis would be provided by retrospective analysis of ataluren clinical trials for treatment of CF nonsense mutations, in which the nonsense mutation within each patient’s CFTR DNA was identified, including whether the mutation was dominant or recessive. Such information, which is not currently in the public domain, would indicate whether there is a correlation between the sequence context dependence of readthrough that we observe (Table 2, Figs 3 and 5–7) and the response of individual CF patients to ataluren treatment. We appreciate the need to protect patient privacy in carrying out such an analysis, but we suggest that the effort required to achieve such protection would be justified, given the potential of a positive result, which would enable pre-selecting CF patients most likely to benefit from ataluren treatment in future trials. The importance of directly linking PTC mRNA sequence context to treatment outcomes is of even greater importance for future trials that use ataluren as part of a combination therapy, and would likely benefit a larger number of CF patients. This number would increase still further if ongoing and future efforts are successful in discovering new TRIDs, which, like ataluren, would exclusively target termination activity at PTCs and retain ataluren’s low toxicity, but with lower EC_50_ values.

## Supplementary Material

gkaf216_Supplemental_Files

## Data Availability

All the materials and methods have been provided in the main text and supplemental information. Further information and requests for resources and reagents should be directed to and will be fulfilled by the corresponding author Barry S. Cooperman (cooprman@pobox.upenn.edu).

## Notes

Present address: Department of Pharmacology and Department of Molecular and Cellular Biology, University of California, Davis, CA 95616, United States

Present address: Department of Pharmacology and Department of Molecular and Cellular Biology, University of California, Davis, CA 95616, United States
