# Supplementary material for: Mechanism-based approach in designing patient-specific combination therapies for nonsense mutation diseases
Source: Nucleic Acids Res. 2025 Mar 29;53(6):gkaf216. doi: 10.1093/nar/gkaf216 (PMC11954524; doi:10.1093/nar/gkaf216)
Supplement: gkaf216_Supplemental_Files [file gkaf216_supplemental_files.zip › Bhat et al, Figure S1.pptx]

## Slide 1
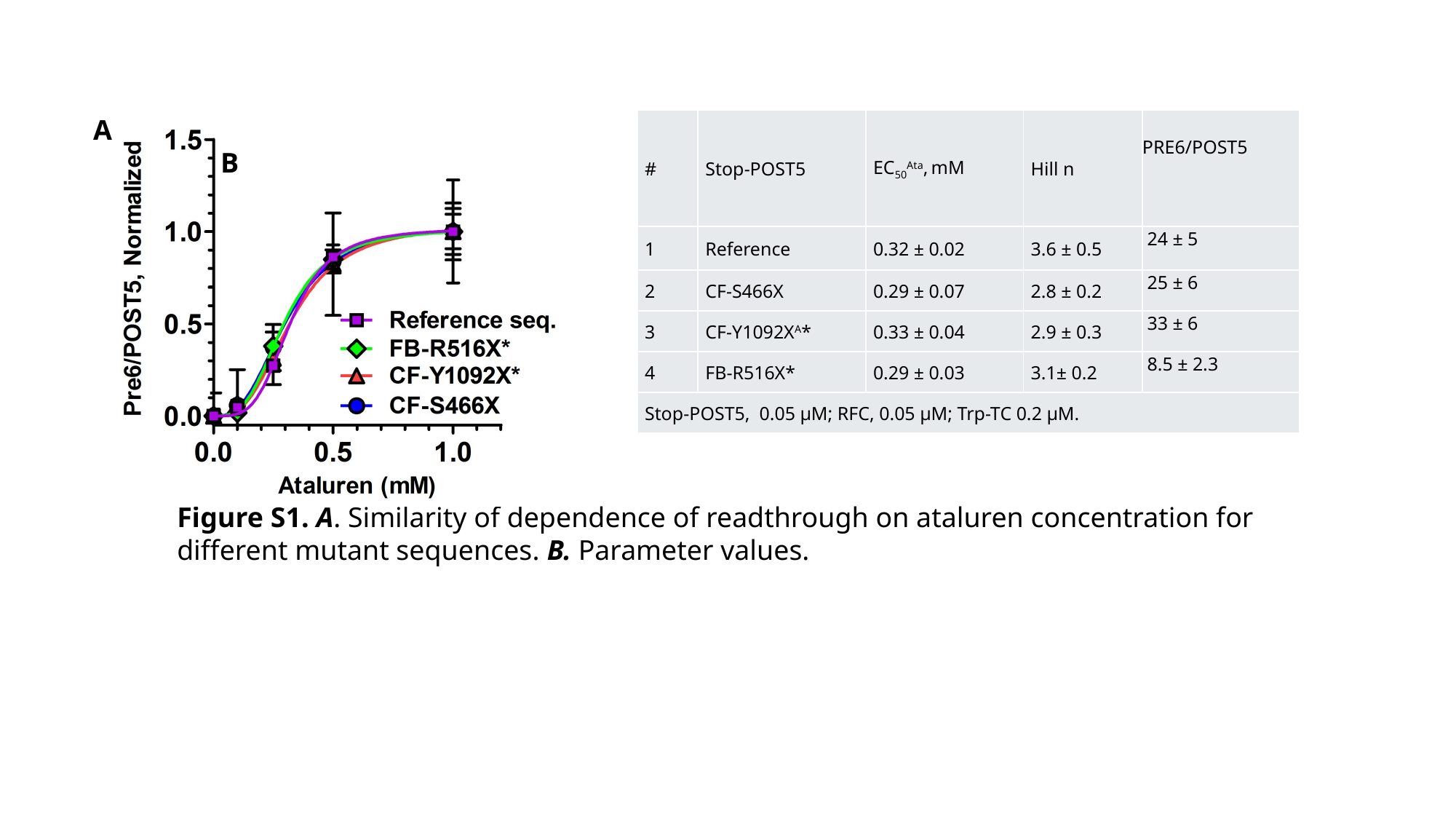

A B
| # | Stop-POST5 | EC50Ata, mM | Hill n | PRE6/POST5 |
| --- | --- | --- | --- | --- |
| 1 | Reference | 0.32 ± 0.02 | 3.6 ± 0.5 | 24 ± 5 |
| 2 | CF-S466X | 0.29 ± 0.07 | 2.8 ± 0.2 | 25 ± 6 |
| 3 | CF-Y1092XA\* | 0.33 ± 0.04 | 2.9 ± 0.3 | 33 ± 6 |
| 4 | FB-R516X\* | 0.29 ± 0.03 | 3.1± 0.2 | 8.5 ± 2.3 |
| Stop-POST5, 0.05 µM; RFC, 0.05 µM; Trp-TC 0.2 µM. | | | | |
Figure S1. A. Similarity of dependence of readthrough on ataluren concentration for different mutant sequences. B. Parameter values.
