# Supplementary material for: Mechanism-based approach in designing patient-specific combination therapies for nonsense mutation diseases
Source: Nucleic Acids Res. 2025 Mar 29;53(6):gkaf216. doi: 10.1093/nar/gkaf216 (PMC11954524; doi:10.1093/nar/gkaf216)
Supplement: gkaf216_Supplemental_Files [file gkaf216_supplemental_files.zip › Bhat et al. - SUPPLEMENTARY INFORMATION .docx]

**SUPPLEMENTARY INFORMATION** included below, consists of Figure S1 and Tables S1 and S2.

| **Table S1. Oligo-DNA sequences used in purifying isoacceptor tRNAs** | | |
| --- | --- | --- |
| tRNA | Codon/Anticodon | Biotin conjugated oligonucleotide sequence |
| Ec-Lys | UUU/AAA | 5- AAA AGT CAA CTG CTC TAC CAA CTG AGC /3Bio/ -3′ |
| Sc-Lys | UUU/AAA | 5- TTA AAA GCC GAA CGC TCT ACC AAC TGA /3Bio/ -3 |
| Ec-Val | GUG/TAC | 5- GTA AGG GAG GTG CTC TCC CAG CTG AGC /3Bio/ -3′ |
| Sc-Val | GUG/TAC | 5- GTG TGA AGG CAA CGT GAT AGC CGC TAC /3Bio/ -3′ |
| Sc-Arg | AGA/1CU | 5- AGA AGT CAG ACG CGT TGC CAT TAC G /3Bio/ -3′ |
| Ec-Gln | CAA/UUG | 5- CAA AAA CCG GTG CCT TAC CGC TTG GCG /3Bio/ -3′ |
| Sc-Gln | CAA/UUG | 5- ATC AAA ACC GAA AGT GAT AAC CAC TAC /3Bio/ -3′ |
| Sc-Trp | UGG/CCA | 5- TTT GGA GTC GAA AGC TCT ACC ATT /3Bio/ -3′ |
| Sc-Leu | CUA/UAG | 5- CTA AAT CTG ACG CCT TAA ACC AC /3Bio/ -3′ |
| Ec-Met | AUG/CAT | 5- ATG AGT GAT GTG CTC TAA CCA ACT GAG C /3Bio/ -3′ |
| Sc-Glu | GAA/UUC | 5′ - GTG AAA GCG TGA TGT GAT AGC CGT TAC /3Bio/ -3′ |
| Sc-Leu | UUG/CAA | 5′ - GCT TGA ATC AGG CGC CTT AGA CCG CTC /3Bio/ -3′ |
| Sc-Thr | ACU/AGU | 5′ - TTA CTA GTG TGG CGC CTT ACC AAC TTG /3Bio/ -3′ |
| Sc-Lys | AAA/UUU | 5′ - TTA AAA GCC GAA CGC TCT ACC AAC TGA /3Bio/ -3′ |
| Sc-Tyr | UAC/GUA | 5′ - ATT ACA GTC TTG CGC CTT AAA CCA ACT /3Bio/ -3′ |
| Ec-Cys | UGC/GCA | 5′ - TGC AAT CCG CTA CAT AAC CGC TTT GTT AAC /3Bio/ -3′ |

| Table S2. Rate constants for RFC interaction with Stop-POST5 complexes determined by smFRET experiments | | | | |
| --- | --- | --- | --- | --- |
| Stop-POST5 | **Sequence downstream** | **k _arrival,app_(min^-1^)** | **k_heRF1 dis_ (min^-1^)** | **k_tRNA dis_ (min^-1^)** |
| Reference | UGA CUA AUG | 1.63 ± 0.14 | 0.061 ± 0.005 | 0.065 ± 0.005 |
| CF-S1196X | UGA CAC GUG | 1.36 ± 0.19 | 0.049 ± 0.006 | 0.062 ± 0.006 |
| CF-W1282X | UGA AGG AAA | 1.50 ± 0.15 | 0.057 ± 0.007 | 0.065 ± 0.006 |
| CF-R1162X | UGA CUC UUU | 1.31 ± 0.18 | 0.039 ± 0.006 | 0.056 ± 0.007 |
| FB-R516X | UGA GCU GGA | 1.56 ± 0.13 | 0.043 ± 0.005 | 0.049 ± 0.005 |
| FB-R2694X | UGA GGA AAC | 1.23 ± 0.17 | 0.045 ± 0.008 | 0.059 ± 0.008 |
| CF-R553X* | UGA GCA AGA | 2.05 ± 0.13 | 0.014 ± 0.003 | 0.012 ± 0.006 |
| CF-RQ30X | UAG CGC CUG | 0.94 ± 0.13 | 0.034 ± 0.006 | 0.064 ± 0.007 |
| CF-Y1092X^G^ | UAG CUG UCA | 1.41 ± 0.12 | 0.048 ± 0.006 | 0.055 ± 0.007 |
| CF-Q685X | UAA UCU UUU | 1.35 ± 0.13 | 0.051 ± 0.006 | 0.052 ± 0.006 |
| CF-E92X | UAA GUC ACC | 1.81 ± 0.14 | 0.038 ± 0.004 | 0.041 ± 0.006 |
